# Measuring community norms around women's empowerment in the West Bank: Opportunities and challenges of a novel approach using cultural consensus

**DOI:** 10.1016/j.ssmph.2019.100489

**Published:** 2019-11-20

**Authors:** Roseanne C. Schuster, Alexandra Brewis, Peggy Ochandarena, Angie Abdelmonem, Sereen Hoso, Khaled Faqeeh

**Affiliations:** a School for Human Evolution and Social Change, Arizona State University, PO Box 872402, Tempe, AZ, 85287-2402, USA; b Global Impact Collaboratory, USA; c Enhanced Palestinian Justice Program, Chemonics International, Ramallah, Palestine; d School for the Future of Innovation in Society, Arizona State University, PO Box 875603, Tempe, AZ, 85287-5603, USA

**Keywords:** Gender, Gender-based violence, Cultural consensus, Culture, Palestine, Development

## Abstract

Understanding cultural norms is essential to achieving results in development interventions and preventing interventions from causing unintended negative consequences. However, capturing norms within everyday contexts in ways that can be monitored and evaluated can be expensive and time consuming and is not always feasible. We tested a novel method, the cultural consensus analysis (CCA), in the context of monitoring and evaluating a United States Agency for International Development (USAID) justice project in the West Bank, Palestine. We conducted 392 survey interviews with men and women, using 60 true or false questions in the knowledge domains of women's empowerment and gender-based violence (GBV), and tested three gender propositions using CCA. We found no singular cultural understanding of women's empowerment and GBV across West Bank Palestinians (proposition 1). Distinctive cultural models for women and other subgroups (e.g., those living in villages, women who identified as discriminated against within Palestinian society) exist, although there were no shared cultural models among men of any subgroup (proposition 2). Program assumptions regarding structural barriers to women's empowerment conformed to the women's cultural models (proposition 3). To our knowledge, this is the first application of CCA as an approach for describing gender norms in international development programming. CCA was able to distinguish subtle cultural patterns, including between population subgroups, and to identify how those are associated with specific risks, such as GBV. We conclude that CCA is a potentially useful approach for development practice, to ground-truth program assumptions and, potentially, to track program impacts.

## Highlights

• Cultural consensus analysis (CCA) is a useful tool to measure norms of women's empowerment in Palestine.
• CCA can be applied to test gender assumptions built into development programs.
• CCA identified deep-set norms but may be less effective at capturing norms in transition.

## Introduction

Ensuring human rights demands attention to gender equity and empowerment. Indeed, women's secondary status remains “one of the true universals, a pan-cultural fact” (Ortner, 1974, p. 67). Gender inequality is perpetuated by cultural norms and practices that normalize women's lower social power and fewer rights and vary in subtle but important ways within communities and across time, relevant to efforts to empower women (Marcus & Harper, 2015). Simultaneously, legal, cultural, and institutional practices can reproduce the gender hierarchies that normalize unequal relations of power between men and women, although the specifics of how this unfolds varies across cultures (Jewkes, Flood, & Lang, 2015).

Despite the long-recognized tenet that gender norms and practices must be carefully attended to for effective international development efforts (Kristjanson et al., 2017), typically, few resources are allocated to identify or confirm them. For example, documents that support development programming can contain assumptive generic cultural statements that are never tested for relevance to the local context. This increases the risk that otherwise technically sound solutions might be rendered ineffective because important cultural norms were not considered or allow for unintentionally negative consequences of development programming for beneficiary populations.

Here we present the results of an application of a novel rapid survey technique—cultural consensus analysis (CCA)—to allow testing of development program assumptions, in the case of a United States Agency for International Development (USAID) project in the West Bank, Palestine. Using CCA, we were able to ground-truth within local communities and in a condensed time frame the assumptions about gender that had been made by development practitioners during the planning phase. For example, do men and women in the West Bank really think differently about gender-based violence (GBV) or women's capacities to advance economically? Do individuals who have experienced gender discrimination espouse different values from others in their local communities who have not experienced it?

Many social science methods that can effectively document such crucial distinctions around gender values within local contexts can be expensive, time consuming, and often logistically difficult to implement (Hanmer & Klugman, 2016). The gold standard is detailed ethnographic data collection (e.g., Inhorn, 2014). In the contexts of the development projects in which we work, time constraints matter; application of a traditional ethnographic approach to capture and explain gender and other cultural norms is not feasible. Our goal, as a collaborative team of anthropologists and development practitioners, was to test the applicability of a different, faster method that anthropologists have been refining for decades but that, to our knowledge, has not been applied to the types of gender assessments conducted in development practice.

CCA captures norm patterns within and across communities. CCA is predicated on cultural consensus theory, which acknowledges that culture is shared knowledge stored in the minds of members of that culture (Romney, Weller, & Batchelder, 1986). CCA can be used to access knowledge in a specific cultural domain, assess variation within that domain and within population subgroups, and identify the most culturally knowledgeable individuals (Hruschka, Sibley, Kalim, & Edonds, 2008). A reasonable public sampling strategy is appropriate to capture key data of interest through rapid survey techniques (Weller, 2007). CCA has been used in this way to extract information about variation in norms across cultures (e.g., body norms and fat stigma; Brewis, Wutich, Falletta-Cowden, & Rodriguez-Soto, 2011) and among subgroups with specialized or divergent knowledge (e.g., birthing practices among skilled attendants vs. lay women; Hruschka et al., 2008), and even to characterize social movements (e.g., Caulkins & Hyatt, 1999).

In cultural consensus surveys, individuals are asked to respond to a set of statements in a prespecified knowledge domain. CCA was initially developed to analyze binary response data (e.g., true or false) and closed multiple choice (Romney et al., 1986), although advancements have been made in analysis for ranked responses (Romney, Batchelder, & Weller, 1987). CCA assumes that respondents correctly answer questions based on a probability of knowing the correct answer and of guessing correctly if they do not know (Weller, 2007). Thus, survey items on cultural norms are meant to be asked in rapid succession to elicit internalized knowledge and minimize second guessing. Data collection for consensus analysis is quite fast (e.g., 20 min per respondent for 60 true/false questions). CCA requires a minimum of 20 items per knowledge domain and 20 respondents per cultural group, assuming moderate agreement within the sample, with more respondents if a wide variation in agreement between respondents is anticipated (Weller, 2007). However, depending on the question of interest, representative sampling on characteristics shown to be influential to the norms in question may be more appropriate. Thus, we tested the proposition that CCA provides a highly cost-effective and efficient method of obtaining information on cultural norms relevant to development programming related to women's empowerment.

### Study context: women, gender, and the enhanced palestinian justice program (EPJP)

In the Middle East and North Africa (MENA), as elsewhere, advancing gender inequality has been a focus for development agencies and civil society organizations. The region has witnessed a long history of local activism challenging gender inequality, much of it tied to national struggles to define the contours of what a modern, postcolonial society should look like (Arenfeldt & Golley, 2012). Since the 1980s, feminist activists have worked in connection with international development organizations to address the status of women and GBV in the MENA, although these efforts have been complicated and shaped, in many ways and various places, by shifting local and global political dynamics over the course of the last century (Abu Lughod, 2013). Authoritarianism, nationalism, liberalism and neoliberalism, globalism, conflict and war, occupation, religious fundamentalism, and development interventions have further impacted the cultural practices that perpetuate gender inequality.

Although gender inequality, cultural practices, and norms are linked, there is nothing particular to the cultures of the MENA that makes gender inequality more exceptional there than elsewhere. Much of the inequality faced by women in the MENA is comparable to that faced by women in other parts of the world. Within the region, there is wide cultural variation, although some general characterizations, such as patriarchal orientation, can be made (Moghadam, 2004). Although there is variability across countries, class, and urbanicity, women in the MENA have less access to education and work, less opportunity to inherit, less access to courts to enforce their rights, and experience higher rates of maternal mortality, bear more responsibility for home care and childcare, are unable to obtain or have difficulty obtaining a divorce, cannot pass nationality on to their children (if married to a foreign man), and participate at lower rates than men in local and national politics (UNDP et al., 2005). Women have fewer legal and political safeguards and options for redress and must negotiate a host of practices and norms regulating their public and private conduct, within governing legal frameworks and beyond the law, which limit their available resources, opportunities, and options (Ahmed Zaki, 2017; Labidi, 2007).

Such gender gaps hold true in the West Bank, where women lack access to and have lower participation in the labor market compared to men, experience discrimination in employment, and lack a comprehensive legal framework for protection (Al-Rifai, Lallement, Said, & Wihaidi, 2013). Similar to elsewhere in MENA, women in the West Bank are expected to prioritize unpaid reproductive and family care roles over a full-time job or profession, and they are further entrenched into these roles by mobility restrictions within the West Bank and a weak social safety net (Al-Rifai et al., 2013). Men continue to be decision-makers within households, even as more women work outside the home—in addition to their caregiving roles—to contribute to family income (Said et al., 2016).

Beyond patriarchal orientation, GBV in the West Bank has been further exacerbated by decades of relentless violence, including economic (sanctions), structural (military occupation), and physical (clashes with Israeli army and settlers) manifestations of violence (Johnson, 2011). The Palestinian Central Bureau of Statistics 2011 survey (the only one in the last 20 years to assess GBV) showed that 35% of married women were exposed to some form of violence by their husband, with 24% subject to physical violence and 12% to sexual violence (PCBS, 2011). Violence based on vulnerable status, including being female, is a starkly evident facet of the sociocultural system in Palestine, which also highly values family honor. Reporting abuses by family members violates social norms, with women and children who report cases of rape or incest likely to incur abuse or even murder by family members because of the shame to family reputation (Elbedour, Abu-Bader, Onwuegbuzie, Abu-Rabia, & El-Aassam, 2006). Furthermore, an offender's marriage to his victim still legally absolves him of rape (Warrick, 2005).

Because of the long-held cultural values and the lack of legal protections from domestic violence, justice sector officials can minimize, ignore, or even punish victims for making complaints. Women are reluctant to report the violence they experience; 65% said they would not discuss the violence, 30% said they would seek assistance from family members, and less than 1% said they would seek assistance from an institution (PCBS, 2011). The lack of a holistic response from the government or nongovernmental organization community worsens the problem. Actors within this space have noted challenges, including insufficient legal and psychosocial services for victims, limited capacity of organizations providing protection services, a dysfunctional national referral system, and problems prosecuting cases because of a slow litigation process and a weak legal framework to protect vulnerable populations who are victims of violence (UNSCO, 2016). Available data are insufficient; for example, women with disabilities are excluded from national statistics. Although there are plans to improve the human rights of vulnerable groups, implementation has lagged. Women's participation in the formal workplace is only 20% and is concentrated in low-paying civil service jobs (42% of civil servants are women), and women are not represented in decision making (representing only 4% of positions in public sector institutions) (PCBS, 2015).

Our study setting is within the context of the USAID-funded EPJP. Implemented by Chemonics International from 2013 to 2018 in the West Bank, EPJP was designed to improve justice sector service delivery by strengthening individual and institutional capacities and increasing citizen engagement with the rule of law. The EPJP theory of change was that more effective and competent justice sector institutions that are accountable to the public and responsive to the needs of citizens would increase citizen satisfaction with Palestinian Authority governance (Chemonics, 2018). EPJP supported development of justice sector policies and procedures, building knowledge and skills of justice sector personnel, renovating and equipping legal institutions, automating processes, and public outreach to increase engagement with the justice system. EPJP also strengthened services to support victims of GBV in government institutions and community sector organizations.

The primary focus of EPJP interventions are the family courts in the West Bank, which have jurisdiction over marriage, divorce, child custody, visitation, and inheritance. Family court judges are traditionally religious leaders rather than lawyers, although this trend is changing. Previously, custom and tradition have been primary factors in judicial decisions. EPJP interventions included training judges to distinguish tradition from law and educating the general population, with a focus on more vulnerable populations, about women's legal rights. Interventions also included educating judges and court staff about GBV and training government and civil society employees (to whom GBV victims are likely to present) on mechanisms to link victims with comprehensive services.

### Study aims: identifying gendered norms using cultural consensus

We use a CCA approach to assess norms among targeted cultural subgroups across the West Bank to test the following propositions: 1) is there a single (shared) Palestinian cultural view on women's empowerment and GBV; 2) are there cultural variations based on gender, age, region, and type of municipality; and 3) do the resulting cultural models accurately reflect the basic assumptions underpinning EPJP gender-based interventions, as reflected in the extended program documents? The approach focuses on assessing ideals of gender empowerment in local cultural terms. Since this was a novel application of the method, our more general goal was to identify possible opportunities, constraints, and solutions to effective application of the method in future development application.

## Methods

### Study design

As is typical in CCA, this study used a cross-sectional design to sample respondents at one time point. However, whereas CCA studies typically sample one characteristic of interest (Alemi, Weller, Montgomery, & James, 2016; Grant; Miller, 2004; Hruschka et al., 2008), we sampled respondents in equal proportions on four key characteristics that may influence cultural beliefs relevant to GBV: gender, age (younger than 35 years, or 35 years and older), type of municipality, and region (Appendix A). The sampling recognized seven key regions of the West Bank: Bethlehem, East Jerusalem, Hebron, Jenin, Nablus, Ramallah, Tulkarem. In each region, 20 participants were to be recruited in each type of municipality (city, village, refugee camp) for a total of 60 participants per region. A city sample was not completed in East Jerusalem because of unrest at the time of data collection.

### Measures

#### Cultural statements

The contents of the cultural consensus tool were developed under the lead of three anthropologists with topical and regional expertise. First, the research team conducted a thorough review of the peer-reviewed and grey literature related to GBV in the Middle East, with a focus on the West Bank, including the intensive gender assessment conducted in an earlier stage of the EPJP (Said et al., 2016). Statements derived from these sources focused on norms propagating GBV and women's economic empowerment in the context of family courts and cultural practices. The research team also reviewed EPJP program documents, including the EPJP contract, first-year work plan, and the USAID/West Bank and Gaza Gender Analysis (Al-Rifai et al., 2013) to identify key program assumptions. These program documents and subsequent statements focused on structural aspects of GBV, including USAID strategic priorities of economic growth and access to public infrastructure, and resulted in statements related to women's understanding of and fulfillment of their rights and cultural and institutional barriers to women's advancement in their careers (e.g., businesses, legal profession, leadership and promotion). A total of 43 agree or disagree statements were initially generated from the literature and program documents.

Meetings were held with the cultural and technical experts to generate more statements. The EPJP team raised the importance of focusing on legal rights related to economic empowerment for divorced women—alimony and child custody—and women receiving inheritances as a driver for GBV. An additional 18 statements were generated from conversations with the EPJP team and further review of the literature. Next a meeting was held with a broader group of technical and cultural program experts, including representatives of community service organizations, that generated an additional 60 statements across the domains of structural dimension of GBV, economic empowerment for women, and household and community dynamics of GBV.

The research team reviewed the 121 generated statements and iteratively refined them into a final total of 60. Very similar statements were collapsed where appropriate. Statements not measuring a clear construct were dropped and statements with compound pieces were broken into two clearer statements. A questionnaire with 70 statements was cognitively tested with three cultural but nontechnical experts. Statements were further revised for clarity, resulting in a total of 64 statements. Trained enumerators then conducted 44 pilot surveys with community members. Challenges with surveys were discussed, and statements were further revised and reduced for conceptual and wording clarity, resulting in the final total of 60 items.

#### Demographics, discrimination, and household decision making

We collected data on key demographics, including gender, age (birth year), marital status, religion, and level of education. To capture additional variables that may be important to analysis, we asked respondents if they had experienced discrimination from Palestinian society and if they had experienced discrimination from their families (never, rarely, sometimes, often, or always).

### Data collection procedures

#### Training

Over two and a half days, the anthropologists trained 20 individuals, including members of the EPJP team and local civil society organizations. Training included interactive discussion on research ethics, cultural consensus theory and models, best practices for survey data collection, and detailed training on this survey protocol and data entry.

#### Sampling

Respondents were recruited from public spaces (e.g., on the street, in shops), because this sampling approach provides a reasonable representation of the group of interest given that cultural values are, by definition, widely shared (Brewis, Gartin, Wutich, & Young, 2013). Effort was made to purposively sample local residents with as broad a range of demographic characteristics as possible. Within each region, an equal proportion of men and women and respondents younger than age 35 and age 35 years and older were recruited from each type of municipality (city, village, refugee camp). This yielded a sample greater than the conservative minimum of 30 respondents per subgroup, based on assumptions of 50% shared beliefs (competency) and high accuracy (0.95) of responses (Weller, 2007).

### Analysis

#### Data cleaning

Responses from paper surveys were entered into an online interface matching the paper survey layout. All data entry was double checked for quality control. Two respondents missing more than 10% of their responses to the cultural statements were dropped, and missing responses for others were randomly imputed according to CCA best practices (Weller, 2007). Four participants were missing key demographic characteristics necessary for testing of subgroups and were also dropped from the sample. Our final sample for analysis was 392 respondents.

#### Consensus analysis

The formal consensus analysis model, using the covariance approach, was run with UCINET software (Borgatti, Everett, & Freeman, 2002). The formal consensus analysis model is analogous to a factor analysis of a respondent-by-respondent correlation matrix—a factor analysis across respondents—that assesses agreement among respondents. This generates factors that represent the pattern of how respondents answer each question and their subsequent agreement with each other.

The first (largest) factor explains as much variation as possible in the respondent correlation matrix and is considered the cultural model (Weller, 2007). A key guideline in determining if a single cultural model exists is if the eigenvalue ratio of the first factor to the second factor is greater than 3.0, indicating that most variability is captured in that cultural model. Conversely, an eigenvalue ratio less than 3.0 may indicate there is not a clear cultural model among that group of respondents, although this is not a strictly observed cutoff.

Each respondent's association or “loading” with the model is that respondent's *cultural competency* score. Loadings or associations with the second factor represent residual agreement between participants not explained by the cultural consensus model. An average cultural competency score greater than 0.5 indicates moderate agreement and greater than 0.66 indicates strong agreement (Brewis et al., 2011). The existence of negative competencies, particularly ones that are non negligibly small (e.g., <-0.1), indicates strong disagreement of the respondent with the cultural model (Borgatti & Halgin, 2011). Second factor loadings measure residual agreement and can be used to identify subgroups (Dressler, Balieiro, & dos Santos, 2015).

Proposition 1 In order to assess existence of one shared cultural model, we conducted consensus analysis on the total sample—all responses to all 60 statements. We checked for an eigenvalue ratio of the first to the second factor greater than 3.0, an average cultural competency score greater than 0.5, and the presence of negative competencies.

Proposition 2 We assessed variations by subgroup, by testing the correlation of the second factor loadings with key sampling and additional characteristics: gender, municipality, region, education (no school, some school, completed high school, or university or technical training), having ever experienced discrimination within the family (no, yes), and having ever experienced discrimination in Palestinian society (no or yes). We used the Pearson correlation for the continuous variable age and its mathematical equivalent eta for categorical variables to test for associations (Alemi et al., 2016; Dressler et al., 2015). If a correlation was significant, we ran additional CCA on the subgroups of that correlation to test the existence of a subcultural model and evaluated model existence using the same parameters in proposition 1.

Proposition 3 We compared the answer key for the 22 statements on structural empowerment derived from program documents with the responses expected based on program documents.

### Ethical statement

Ethics oversight and approval for the study was provided by the Arizona State University Institutional Review Board.

## Results

Respondents were on average 36.5 years old, married (64%), and well educated, with 56% having university or technical training and 27% a high school diploma (Table 1). Women were more likely to be divorced or widowed than men in the sample, and women and individuals living in cities had higher levels of education compared to other subgroups. Men reported greater frequencies of discrimination from Palestinian society than women, and individuals living in cities reported less frequent societal discrimination than those in villages or refugee camps.

**Table 1:** Demographic characteristics of respondents to a cultural survey on gender-based violence in the West Bank, Palestine.

Characteristic	Total (n = 392)	Men (n = 195)	Women (n = 197)	*Gender p-*value	City (n = 117)	Village (n = 139)	Refugee Camp (n = 136)	*Municipality p-*value
**Age** Mean + SD (years)	36.5 ± 11.7	37.1 ± 11.6	36.0 ± 11.8	*0.254*	35.0 ± 10.7	36.3 ± 11.8	38.1 ± 12.3	*0.105*
**Location**				*0.847*				
City	117 (29.8%)	56 (28.7%)	61 (31.0%)					
Village	139 (35.5%)	71 (36.4%)	68 (34.5%)					
Refugee camp	136 (34.7%)	68 (34.9%)	68 (34.5%)					
**Region**				*0.942*				*0.082*
Bethlehem	55 (14%)	30 (15.4%)	25 (12.7%)		17 (14.5%)	18 (12.9%)	20 (14.7%)	
E Jerusalem	39 (9.9%)	20 (10.3%)	19 (9.6%)		0 (0.0%)	20 (14.4%)	19 (14.0%)	
Hebron	61 (15.6%)	30 (15.4%)	31 (15.7%)		20 (17.1%)	21 (15.1%)	20 (14.7%)	
Jenin	60 (15.3%)	26 (13.3%)	34 (17.3%)		20 (17.1%)	22 (15.8%)	18 (13.2%)	
Nablus	59 (15.1%)	29 (14.9%)	30 (15.2%)		20 (17.1%)	19 (13.7%)	20 (14.7%)	
Ramallah	59 (15.1%)	31 (15.9%)	28 (14.2%)		21 (17.9%)	19 (13.7%)	19 (14.0%)	
Tulkarem	59 (15.1%)	29 (14.9%)	30 (15.2%)		19 (16.2%)	20 (14.4%)	20 (14.7%)	
**Marital status**				*0.003*				*0.412*
Single	118 (30.2%)	57 (29.4%)	61 (31.0%)		38 (32.5%)	39 (28.3%)	41 (30.1%)	
Married	250 (63.9%)	134 (69.1%)	116 (58.9%)		77 (65.8%)	89 (64.5%)	84 (61.8%)	
Divorced	10 (2.6%)	2 (1.0%)	8 (4.1%)		1 (0.9%)	5 (3.6%)	4 (2.9%)	
Widowed	13 (3.3%)	1 (0.5%)	12 (6.1%)		1 (0.9%)	5 (3.6%)	7 (5.1%)	
**Education**				*0.002*				*0.004*
No School	6 (1.6%)	6 (3.1%)	0 (0.0%)		1 (0.9%)	1 (0.7%)	3 (2.2%)	
Some school	63 (16.3%)	35 (18.3%)	28 (14.3%)		11 (9.5%)	22 (16.1%)	30 (22.1%)	
Completed high school	103 (26.6%)	60 (31.4%)	43 (21.9%)		24 (20.7%)	36 (26.3%)	34 (25%)	
University/technical training	215 (55.6%)	90 (47.1%)	125 (63.8%)		80 (69.0%)	76 (55.5%)	59 (43.4%)	
**Religion**				*0.135*				*0.076*
Muslim	385 (99.0%)	192 (99.0%)	193 (99%)		114 (98.3%)	137 (100%)	134 (98.5%)	
Christian	2 (0.5%)	0 (0.0%)	2 (1.0%)		2 (1.7%)	0 (0.0%)	0 (0.0%)	
Other	2 (0.5%)	2 (1.0%)	0 (0.0%)		0 (0.0%)	0 (0.0%)	2 (1.5%)	
**Discriminated against/mistreated by Palestinian society**				*0.038*				*0.029*
Never	202 (51.7%)	104 (53.3%)	98 (50.0%)		74 (63.2%)	68 (48.9%)	60 (44.4%)	
Rarely	141 (36.1%)	66 (33.8%)	75 (38.3%)		36 (30.8%)	54 (38.8%)	51 (37.8%)	
Sometimes	30 (7.7%)	11 (5.6%)	19 (9.7%)		5 (4.3%)	9 (6.5%)	16 (11.9%)	
Often or always	18 (4.6%)	14 (7.2%)	4 (2.0%)		2 (1.7%)	8 (5.8%)	8 (5.9%)	
**Discriminated against/mistreated by family**				*0.063*				*0.344*
Never	287 (74.5%)	149 (77.6%)	138 (71.5%)		83 (72.2%)	108 (79.4%)	96 (71.6%)	
Rarely	74 (19.2%)	34 (17.7%)	40 (20.7%)		28 (24.3%)	19 (14.0%)	27 (20.1%)	
Sometimes	15 (3.9%)	3 (1.6%)	12 (6.2%)		2 (1.7%)	6 (4.4%)	7 (5.2%)	
Often or always	9 (2.3%)	6 (3.1%)	3 (1.6%)		2 (1.7%)	3 (2.2%)	4 (3.0%)	

### Proposition 1. Existence of a single (shared) Palestinian cultural view on GBV

There was no single model of the cultural norms surrounding women's empowerment and GBV among the West Bank Palestinians sampled, as indicated by the low eigenvalue ratio (2.4), large number of negative cultural competencies (36; 11 nonnegligible), and low average competency score (0.32).

### Proposition 2. Detecting important variations based on gender, age, region, and type of municipality

Second factor loadings for all respondents correlated significantly with gender (*eta* = 0.232, p < .001), region (*eta* = 0.202, p = .01), having experienced discrimination by Palestinian society (*eta* = 0.152, p = .003), and having experienced discrimination from family members (*eta* = 0.190, p < .001). We divided respondents into corresponding subgroups for each of these characteristics (e.g., men and women; each of the seven regions) and conducted CCA. Although not significantly correlated to the second factor loadings for all respondents, we also ran CCA according to municipality subgroups, as this was a key sampling frame.

From these subgroups, we present model parameters for subgroups with eigenvalue ratios greater than the guideline of 3.0 (Table 2), although average cultural competency scores for these models were below the threshold for moderate agreement of 0.5. By gender, women showed a distinct cultural understanding as demonstrated by an eigenvalue ratio above 3.0 but a lower-than-moderate average competency score (0.37) and several negative competencies (3 nonnegligible). Men did not meet the eigenvalue ratio guideline, indicating no agreement. Second factor loadings for men's and women's models were significantly correlated with having experienced discrimination within society (men, *eta* = 0.145, *p* = .04; women *eta* = 0.219, *p* = .002) and within the family (men, *eta* = 0.158, *p* = .028; women, *eta* = 0.173, *p* = .015); therefore, we ran CCA on these subgroups. Only models for women were distinct: women who have or have not experienced discrimination in Palestinian society and women who have not experienced discrimination from their families. There was no agreement, supported by lack of cultural models, among men by any subgroup.^1^

**Table 2:** Parameters for cultural models among key subgroups surrounding women's empowerment and gender-based violence in the West Bank, Palestine.

		Region		Discrimination			Municipality		
	Women	East Jerusalem	Nablus	Women -Never experienced within Palestinian society	Women – Experienced within Palestinian society	Women - Never experienced within family	Residents of villages	Women in villages	Women in refugee camps
n	197	61	59	98	98	138	**139**	**68**	**68**
Eigen ratio	3.016	3.689	3.287	3.013	3.153	3.091	3.118	3.663	3.622
Number of negative com-petencies (non-negligible)	14 (3)	1 (1)	1 (1)	10 (2)	4 (1)	10(2)	9 (3)	1 (1)	6(2)
Mean competency score	0.372	0.406	0.433	0.358	0.392	0.365	0.355	0.417	0.277

**Table 3:** Answer key for most comprehensive cultural model (all women) for women's empowerment and gender-based violence in the West Bank, Palestine.

Items	All women (n = 197)
**Structural Empowerment (Program Assumptions)**	
Women have more opportunities in society than men	No
Men and women have equal opportunities to earn money in Palestine	No
Women have the same legal guardianship of their children as men do	No
Women do **not** have the same rights and legal protection as men do.	Yes
Only husbands can speak to their wives as they please.	No
Women today face a lot of pressure to earn some money **and** do most of the household work	Yes
Women have a more difficult time than men moving around in my community	Yes
There are few women in public office because few women are as qualified as the men	No
Having more women leaders in legal system will lead to changes in how women are treated by the law	Yes
Women who start businesses have more difficulty getting bank capital due to cultural norms	No
Women feel their skills are inadequate to start a business	No
Women are just as likely to be promoted to senior management positions as men who have the same qualification	No
Having women participate in community meetings is limited due to the timing of these meetings	Yes
Early marriage leads girls to drop out of secondary school	Yes
Women have a clear understanding of their legal rights	No
Women are discouraged from accessing formal legal services by regulations and policies	Yes
Increasing public awareness about sexual harassment and discrimination towards women may increase women's likelihood to access justice	Yes
Women want more information on the right and responsibilities of men towards them	Yes
Women are not interested in jobs with leadership roles	No
Men do not want women in leadership roles	Yes
When women hear about the experiences of successful female leaders they will be inspired to seek out these positions	Yes
The i ncrease of a woman's awareness of her rights will lead to an increase in violence against her	Yes
**Economic Empowerment**	
Women avoid the courts for family matters because they are unfair to them	No
Cultural norms make it difficult for a woman to bring a case against her relatives	Yes
Women are always given their fair share of inheritance with their brothers	No
To give any inheritance to a woman is a generosity/kindness from her family	No
A woman with her own money has too much power	Yes
A woman would avoid court to solve family issues (like inheritance) because it takes too long	Yes
Women do not ask for their inheritance to avoid violence against her from her family	Yes
A woman who gets substantial alimony in a divorce has a much better life	Yes
A woman who gets her inheritance through family court, she is more likely to get hit or hurt	Yes
It is good for society that men and women get inheritance share	Yes
Women get better alimony by accessing formal family court than the tribal system	Yes
Only men make the decisions about how inheritances are distributed	No
Women who marry early are more likely to divorce	Yes
Women should not inherit any property because it will go to her husband's family	No
Generally, men think they have the right to control women	Yes
Men have the right to make decisions more than women	Yes
Women are treated fairly in the family court	Yes
Women deserve equal custody of children after divorce, regardless of their children's age	Yes
**Gender-based violence**	
Women who have been hit by their husbands hit their children	No
Women victims prefer to stay silent	Yes
Women endure domestic violence to keep the family together	Yes
Women who act and dress seductively are usually the women who get raped	Yes
Men hurt women because they can	Yes
If a woman contributes financially to her household she takes power away from man	No
Poverty leads to increased violence in the household	Yes
Stressed men are more likely to hit their wives	Yes
In an extended family, women cannot make any decisions for themselves	Yes
All the men in the family can use violence to teach a woman how to behave	No
When a rapist marries his victim, it fixes the situation in the eyes of the community	Yes
Family members often cut off relationships with a woman seen as regularly disobeying men in her household	Yes
It is acceptable for a woman to seek protection outside of the household if she feels threatened	No
Most women blame themselves for violence they experience	Yes
Women who feel threatened by their family seek help outside their household	Yes
A woman experiencing violence in her household can do more domestic chores in hope this will stop the violence	Yes
The police will not help women experiencing violence in the household	No
Women's institutions will help women experiencing violence in the household	Yes
A woman who has sex outside of marriage brings shame on her family	Yes
A family that kills a sexually improper woman has reclaimed its honor	No

By region, respondents in East Jerusalem and Nabulus were the only regions with a shared cultural model. Average cultural competency scores were still below moderate agreement (0.406 and 0.433, respectively), although higher than in other subgroups. None of the second factor loadings for any of the seven regions significantly correlated with additional characteristics (e.g., gender, education, age, municipality) to suggest further subgroups.

By municipality, only respondents in villages showed a shared understanding of norms around gender empowerment and GBV. Testing second factor loadings for each of cities, villages, and refugee camps showed correlations with gender only. Testing these subgroups further revealed shared cultural understandings among women in villages, and although eigenvalue ratio criteria were met for women in refugee camps, average cultural competency was very low (0.278).

No subgroups of men or individuals living in cities, or any subgroups within these characteristics, met any of the model parameters, suggesting greater disagreement among respondents with these characteristics.

### Beliefs about gender empowerment

#### Structural barriers

Broadly, the cultural models recognized that women do not have equal opportunities, legal rights, or mobility in their communities compared with men and are disadvantaged in terms of education and legal guardianship of children. The models affirm that women encounter growing pressure to maintain traditional roles while increasing contributions to household income and challenges in advancing their careers in terms of promotion and leadership. The Palestinians sampled—except those living in East Jerusalem, the only region in the sample operating under Israeli law—agree that the current gap is not due to poorly qualified or uninterested women and that having more women in the legal profession would lead to more equitable laws.

#### Economic empowerment

All subgroups held the norm that women are treated fairly in family court, but women who have themselves experienced discrimination believe that women avoid family courts because they *perceive* them to be unfair. Subgroups believe women avoid courts because the process takes too long, that cultural norms make it difficult for a woman to bring a case against her relatives, and that women get better alimony through family court than tribal arbitration.

All subgroups believe that men think they have the right to control women and most—except those in Nablus and women who have never experienced discrimination within the family—hold the norm that men have the right to make decisions more than women. In all subgroups except those from East Jerusalem, respondents do not believe that only men make decisions about distribution of inheritances. Subgroups believe that a woman with her own money has too much power but that a woman's financial contributions to the household do not take power away from a man; this may be linked to the norm that there is more pressure for women to contribute financially and continue to do all domestic work. Although most subgroups agree that women cannot make decisions for themselves in extended families, women who have never experienced discrimination in Palestinian society disagree with that norm.

Norms across subgroups were that although beneficial, women typically do not receive their legally allotted inheritance, do not ask for their inheritance in order to avoid violence, and are more likely to endure physical violence if they receive inheritance through family court. Most subgroups—except women who have never experienced discrimination in Palestinian society and individuals in East Jerusalem—reported that women will experience increasing violence with increasing awareness about their rights.

#### Gender-based violence

Subgroups endorsed the norms that men hurt women because they can, that stress and poverty lead to increased violence, and that GBV victims often stay silent, endure violence in order to keep their families together, and hope that increased domestic diligence will stop the violence. Except for those living in villages, subgroups believe most women blame themselves for violence they experience. Subgroups of women who have experienced discrimination in society and respondents living in East Jerusalem agreed that abused women are more likely to hit their children.

All cultural models report that it is the norm for family members to sever relationships with a woman perceived to regularly disobey men in her family and that although women who feel threatened do seek help outside her household, this is not socially acceptable. It is the norm that non-profits supporting women and police will help women in the West Bank who have experienced domestic violence—except in East Jerusalem, which is under Israeli police. Subgroups held the norms that women who have sex outside of marriage bring shame upon their families, that women who act and dress immodestly are often rape victims, and that marrying one's rapist fixes the wrong.

### Proposition 3. Assessing whether cultural models reflect EPJP program assumptions

Program assumptions about cultural norms of structural factors were predominantly validated by the existing cultural consensus models among women and further subgroups of women, individuals in East Jerusalem, and individuals living in Nablus. There were two statements in which the answer for cultural models based on 22 items differed from program expectations. First, although most subgroups do not believe that women starting businesses have more difficulty obtaining bank capital, subgroups of women who have experienced societal discrimination and residents of East Jerusalem agreed this is the norm. Second, all subgroups disagreed with the statement that “women feel their skills are inadequate to start a business.” This suggests that in contrast to the gender assessment, communities believe women feel they have the skills to start a business, so other barriers may be more pressing.

## Discussion: opportunity and challenges in using CCA for gender assessments

Using cultural consensus analysis, our goal was to identify variation in cultural norms surrounding gender empowerment and GBV in the West Bank. We have identified that there is not a single cultural model of understanding. Furthermore, although models among subgroups captured greater variability, we did not find moderate or strong agreement as measured by the cultural competency score within key subgroups (women, respondents in villages, women in villages, women in refugee camps, women who have/have not experienced discrimination within society, women who have not experienced discrimination within their families, and respondents living in the regions of Nablus and East Jerusalem).

These findings suggest wide variation in West Bank Palestinians' understanding of cultural norms surrounding women's empowerment. This may be driven by the shifting status of some gender norms in the West Bank. Education and wealth are driving shifts in gender norms in the MENA region more broadly (Abu Rabia Quedar, 2007). Palestinian women are among the most highly education in the MENA but participate in the labor market at lower rates (Fawcett, 2018). Although more women in the West Bank are working outside the home than in previous decades, the money they earn often goes to support their families and may not lead to personal autonomy, which is already a contested concept as the identity of women in the MENA is tightly intertwined with familial roles (Ahmed, 2018). It is worth noting that women in our sample were more highly educated than the national average (Fawcett, 2018) and that we did not find shared cultural models by education; however, our highest education category collapsed together university and technical training, which includes basic-to-specialized training, and may have obscured differences.

From a programmatic perspective, the assertion of likelihood of violent and unintended consequences for women claiming their rights in divorce and inheritance is striking. EPJP has trained judges on the laws regarding women's rights in these contexts and conducted public outreach, including events where judges and other legal partners answer women's situational questions. However, the risk for violence when women claim their rights emphasizes the need for targeted programming and education of the men and women who are part of the system by which structural and physical violence is replicated (e.g., decision makers around inheritance), to avoid these explicit and unintended consequences.

This study affirmed that the norms propagating GBV that are true in many communities across the world exist in the West Bank as well, such as how poverty and stress are correlated with domestic violence (Fahmy, Williamson, & Pantazis, 2016). GBV norms more specific to the MENA region were also affirmed, including marriage fixing the wrongs of rape and ostracization of women who regularly disobey men by family members (Warrick, 2005). To the extent that the lack of strong agreement suggests that norms are changing, this is hopeful from a women's empowerment perspective—although there is still a long way to go.

### Challenges to use of cultural consensus analysis

These findings suggest that rather than identifying cultural models, identifying lack of a cultural model and agreement may be more important in knowledge domains in which cultural norms are rapidly shifting and vary among many subgroups. CCA has been important in discerning between models of cultural understanding among arguably more static subgroups with clear differences in training related to that domain (e.g., traditional birth attendants, lay women, and skilled birth attendants; Hruschka et al., 2008), experience (e.g., local community members and conservation officers, Grant & Miller, 2004), and comparisons across national cultural contexts (Brewis et al., 2011, 2013; Crona et al., 2013). However, the increasing activity towards women's empowerment in the West Bank, against a backdrop of Israeli occupation and pervasive paternalistic culture, creates pressures with contours that likely vary by subgroup. We found low agreement on cultural models of women's empowerment and GBV, divided along key population characteristics of gender, variation in regional governance (e.g., East Jerusalem being governed by Israeli law), and lived experiences of discrimination. Strikingly, we found no evidence of shared understanding among a major subgroup—men, although the sample size (n = 195) would have been sufficient assuming conservative levels of competency (Weller, 2007). Correctly identifying results such as these—that knowledge domains may be contested or shifting and that subgroups lack agreement—may be a relevant challenge for use of this approach in development practice.

Another challenge to development practitioners’ uptake of this method is the specialized knowledge required and supplied by the anthropologist collaborators of this study. The knowledge of cultural consensus theory, the art of and time spent on creating questions around norms, and the analysis required may extend beyond the standard skill set for monitoring and evaluation specialists. Thus, cost efficiencies realized through the data collection method (e.g., lower sample size requirements than typical randomized household surveys, rapid survey deployment) may be offset by costs incurred in expertise to support program personnel, which may only be available outside the country or region. We hope that increased capacity in CCA will help to mitigate this challenge.

### Opportunities presented by cultural consensus analysis

These findings show that CCA presents an opportunity to rapidly collect data on cultural norms that can inform gender programming. Our findings of overall agreement with key program assumptions suggest CCA provides an efficient means to ground-truth basic program assumptions as reflected in the documents and discussions that are key in intervention planning and designs.

From a programmatic standpoint, the low agreement among subgroups suggests CCA may sometimes be just a first step, with more detailed study necessary to understand attitudes towards women's empowerment within key subgroups. But CCA does help identify exactly which subgroups those should be. Methodologically, it is unusual that weak agreement would be present among subgroups with a distinct understanding as measured by an appropriate eigenvalue ratio. It is possible that other factors affecting respondents' intersectionality of identity may not have been measured in this study and we were unable to extract them, although advances in alternative CCA procedure could help (Anders, Alario, & Batchelder, 2017). For example, the lack of any evidence of a shared cultural model among men suggests that further qualitative work with men to elicit their understanding of women's empowerment may be useful in targeting programming towards men to shift these norms.

### Limitations

Although we designed the statements to achieve a balanced design, e.g., an even split between agree and disagree responses, the answer key identifies close to two-thirds of the 60 statements respondents agreed with. However, as the formal model is not sensitive to response bias but rather to all “true” or all “false” statements (Romney et al., 1986), this is not a limitation.

Our study was a pilot, using a generally unproven method for working in these development contexts. Our survey items (i.e., cultural statements) were derived from thorough literature review and underwent a rigorous creation and revision process with local experts and sought to test assumptions made in program documents. However, this approach contrasts the emic origins of the fields of cognitive science from which cultural consensus emerged. Thus, our approach introduces potential limitations: that the identified domains may not have been sufficiently coherent or explicit and the questions not sufficiently direct in the way people think and speak about GBV. Cultural domain analyses (e.g., pile sorts, free lists) are rapid ethnographic methods that may be used to identify or confirm appropriate domains.

## Conclusions

Our study identifies potential utilities of CCA to rapidly identify meaningfully local variation in norms around gender empowerment and to validate gender assumptions in program design. To our knowledge, this is the first use of CCA in the context of international development program assessment. Our sample size was larger and purposively more diverse than is usual with CCA studies, and this allowed testing of cultural models within multiple subgroups. The diversity of norms within subgroups—particularly among men—once identified could be used to better design, target, and monitor empowerment interventions that support women's shared ideals. The results are sufficiently compelling to encourage further testing of this method within gender and development activities. By applying the method to other topics in other settings, it will be easier to identify how and when this novel approach might be most reliable, valid, and useful. Ultimately, better methods to rapidly and efficiently appraise cultural knowledge and to identify related cultural barriers, with respect to gender (or other) programming, would provide a major enhancement to development practice.

## Financial disclosure

The Enhanced Palestinian Justice Program was funded by the United States Agency for International Development (Contract No. 294-C-13-00006-00) to Chemonics International. The Global Impact Collaboratory (RCS, AB, PO) received support for this study from Arizona State University International Development, USA and Chemonics International, Inc, USA. AA is supported by a National Science Foundation Directorate for Social, Behavioral, and Economic Sciences Postdoctoral Research Fellowship (grant number 1714864), USA.

## Declaration of competing interest

Authors have no conflicts of interest to report.

## Figures and Tables

**Figure:**
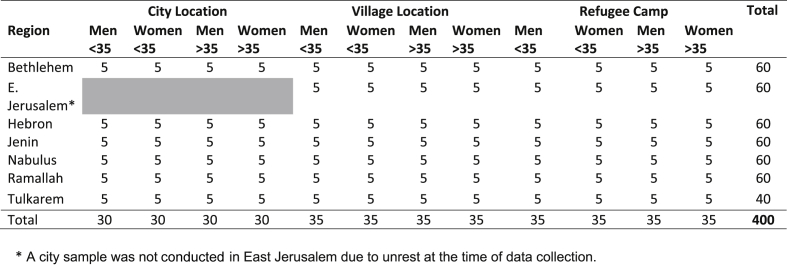


## Footnotes

1 Due to concerns about the persistent nature of residual agreement within cultural consensus, we conducted a second approach based on Henderson and Dressler (2017). After testing and failing to identify the existence of a single cultural model, we split the total sample into thirds based on residual agreement (second factor loadings). Using chi-square, we identified that those in the subgroup with the lowest residual scores were significantly more likely to be men, living in Nablus, and had never experienced discrimination by their families or Palestinian society. Those with the highest residual scores were significantly more likely to be women, living in East Jerusalem, and had experienced discrimination by their families and Palestinian society. For these subgroups with the highest and lowest residual agreements, we calculated mean deviation from the degree of consensus, based on the weighted average of the UCINET output from the cultural consensus command per each item. Then we plotted the deviation from degree of consensus per each item for the subgroup with the highest residual scores against the subgroup with the lowest residual scores. The correlation for the entire sample was −0.4586 (*p* = .002), suggesting that several items endorsed by one group were less likely to be endorsed by the other group. After testing and identifying the existence of one cultural model for all **women**, we again split the sample into thirds by residual scores. Using chi-square, we identified that women in the subgroup with the lowest residual scores were significantly more likely to have never experienced discrimination by their families or Palestinian society and those with the highest residual scores were significantly more likely to have experienced discrimination by both. We used the same procedure to calculate deviation of the subgroups from the degree of consensus (weighted mean) and plotted these against each other. The marginally significant negative correlation of −0.2477 (*p* = .056) suggests that these subgroups, differentiated by experiences of discrimination, differentially prioritized certain statements. After testing and failing to identify the existence of one cultural model for all **men**, we followed the same procedure. Using chi-square, we identified that men in the subgroup with the lowest residual scores were significantly less likely to be from East Jerusalem. The significant negative correlation of −0.6876 *(p*=<.0001) again suggests that several statements endorsed by one group were less likely to be endorsed by the other group.

## References

[bib1] Abu Lughod L. (2013). *Do Muslim women need saving*?.

[bib2] Abu Rabia Quedar S., Maghadam (2007). Education, tradition, and modernization: Bedouin girls in Israel. From patriarchy to empowerment: Women's participation, empowerment, and rights in the Middle East, North Africa, and South Asia.

[bib3] Ahmed G. (2018). Someone sleeping in your bed: On feminism and marriage in Egypt, Mada. https://www.madamasr.com/en/2018/08/18/feature/society/someone-sleeping-in-your-bed-on-feminism-and-marriage-in-egypt/.

[bib4] Ahmed Zaki H. (2017). “Law, culture, and mobilization: Legal pluralism and women's access to divorce. Muslim World Journal of Human Rights.

[bib5] Al-Rifai A., Lallement D., Said N., Wihaidi R. (2013). USAID/West Bank and Gaza Gender analysis.

[bib6] Alemi Q., Weller S.C., Montgomery S., James S. (2016). Afghan refugee explanatory models of depression: Exploring core cultural beliefs and gender variations. Medical Anthropology Quarterly.

[bib7] Anders R., Alario F.X., Batchelder W.H. (2017). Consensus analysis for populations with latent subgroups: Applying multicultural consensus. Cross-Cultural Research.

[bib8] Arenfedlt P., Al-Hassan Golley N. (2012). Mapping Arab women's movements: A century of transformations from within.

[bib9] Borgatti S.P., Everett M.G., Freeman L.C. (2002). Ucinet for windows: Software for social network analysis.

[bib10] Borgatti S.P., Halgin D., Kronenfeld D., DeMunck V., Fischer M., Bennardo G. (2011). Consensus analysis. Blackwell's companion to cognitive anthropology.

[bib11] Brewis A., Gartin M., Wutich A., Young A. (2013). Global convergence in ethnotheories of water and disease. Global Public Health.

[bib12] Brewis A., Wutich W., Falletta-Cowden A., Rodriguez-Soto I. (2011). Body norms and fat stigma in global perspective. Current Anthropology.

[bib14] Caulkins D., Hyatt S. (1999). Using consensus analysis to measure cultural diversity in organizations and social movements. Field Methods.

[bib15] Chemonics International. EPJP first year workplan: October 1, 2013 – September 30, 2014.

[bib16] Chemonics International (2018). Enhancing Palestinian Justice Program final report.

[bib17] Crona B., Wutich A., Brewis A., Gartin M. (2013). Perceptions of climate change: Linking local and global perceptions through cultural knowledge. Climatic Change.

[bib18] Dressler W.D., Balieiro M.C., dos Santos J.E. (2015). Finding culture change in the second factor: Stability and change in cultural consensus and residual agreement. Field Methods.

[bib19] Elbedour S., Abu-Bader S., Onwuegbuzie A.J., Abu-Rabia A., El-Aassam S. (2006). The scope of sexual, physical, and psychological abuse in a Bedouin-Arab community of female adolescents: The interplay of racism, urbanization, polygamy, family honor, and the social marginalization of women. Child Abuse & Neglect.

[bib20] Fahmy E., Williamson E., Pantazis C. (2016). Evidence and policy review: Domestic violence and poverty.

[bib22] Fawcett H. (2018). Highly educated Palestinian women struggle to find jobs. https://www.aljazeera.com/news/2018/03/highly-educated-palestinian-women-struggle-find-jobs-180310131613369.html.

[bib23] Grant K.L., Miller M.L. (2004). A cultural consensus analysis of marine ecological knowledge in the Solomon Islands. SPC Traditional Marine Resource Management and Knowledge Information Bulletin.

[bib24] Hanmer L., Klugman J. (2016). Exploring women's agency and empowerment in developing countries: Where do we stand?. Feminist Economics.

[bib25] Henderson N.L., Dressler W.W. (2017). Medical disease or moral defect? Stigma attribution and cultural models of addiction causality in a university population. Culture Medicine and Psychiatry.

[bib27] Hruschka D.J., Sibley L.M., Kalim N., Edonds J.K. (2008). When there is more than one answer key: Cultural theories of postpartum hemorrhage in Matlab, Bangladesh. Field Methods.

[bib28] Inhorn M.C. (2014). Roads less traveled in Middle East anthropology—and new paths in gender ethnography. Journal of Middle East Women's Studies.

[bib29] Jewkes R., Flood M., Lang J. (2015). From work with men and boys to changes of social norms and reduction of inequities in gender relations: A conceptual shift in prevention of violence against women and girls. Lancet.

[bib30] Johnson P. (2011). Violence, gender-based violence, and protection: A dangerous decade. A dangerous decade: The 2^nd^ gender profile of the occupied West Bank and Gaza (2000-2010).

[bib31] Kristjanson P., Bryan E., Bernier Q., Twyman J., Meinzen-Dick R., Kieran C. (2017). Addressing gender in agricultural research for development in the face of a changing climate: Where are we and where should we be going?. International Journal of Agricultural Sustainability.

[bib32] Labidi L., Moghadam V.M. (2007). Islamic law, feminism, and family. In from patriarchy to empowerment: Women's participation, movements, and rights in the Middle East, North Africa, and South Asia.

[bib34] Marcus R., Harper C. (2015). How do gender norms change? Knowledge to action resource series. UKAID. Knowledge to action resource series.

[bib35] Moghadem V. (2004). Patriarchy in transition: Women and the changing family in the Middle East. Journal of Comparative Family Studies.

[bib36] Ortner S.B., Rosaldo M.Z., Lamphere L. (1974). Is female to nature and male is to culture?. In *women, culture, and society*.

[bib37] Palestinian Central Bureau of Statistics (PCBS) (2011). Main findings of violence survey in the Palestinian society, 2011. Ramallah - Palestine. http://www.pcbs.gov.ps/portals/_pcbs/pressrelease/el3onfnewenglish.pdf.

[bib38] PCBS (2015). Labour force survey results: April –June, 2015 Round (Q2/2015). Results: Ramallah, Palestine. http://www.pcbs.gov.ps/portals/_pcbs/PressRelease/Press_En_english%20press.pdf.

[bib40] Romney A.K., Batchelder W.H., Weller S. (1987). American Behavioral Scientist.

[bib41] Romney A.K., Weller S.C., Batchelder W.H. (1986). American Anthropologist, New Series.

[bib42] Said N., Ziad-Ghattas R., Al-Araj N., Shuaibi M., Faraj S., Shukri V. (2016). Comprehensive analysis for gender-based violence and the status of the national referral system in West Bank.

[bib43] UNDP, Arab Fund for Economic and Social Development, Arab Gulf Programme for United Nations Development Organizations (2005). Arab human development report: Towards the rise of women in the Arab world.

[bib44] United Nations Special Coordinator for the Middle East Peace Process (UNSCO) (2016). Leave no one behind: A perspective on vulnerability and structural disadvantage in Palestine. https://www.un.org/unispal/document/leave-no-one-behind-a-perspective-on-vulnerability-and-structural-disadvantage-in-palestine-unct-report/.

[bib46] Warrick C. (2005). The vanishing victim: Criminal law and gender in Jordan. Law & Society Review.

[bib47] Weller S. (2007). Cultural consensus theory: Applications and frequently asked questions. Field Methods.

